# Effect of adalimumab on choroidal thickness and choroidal vascularity index in eyes with non-infectious uveitis using enhanced-depth imaging optical coherence tomography

**DOI:** 10.1038/s41433-024-02975-9

**Published:** 2024-02-20

**Authors:** Cem Evereklioglu, Elif Er Arslantas, Hidayet Sener, Zeynep Akkul, Kamran Gahramanov, Duygu Gulmez Sevim, Osman Ahmet Polat, Fatih Horozoglu

**Affiliations:** Department of Ophthalmology, Division of Uvea-Behçet Unit, Erciyes University Medical Faculty, Kayseri, Türkiye

**Keywords:** Diseases, Pathogenesis

## Abstract

**Objective:**

To evaluate the effect of adalimumab (ADA) on choroidal thickness (ChT) and choroidal vascularity index (CVI) in eyes with non-infectious uveitis (NIU).

**Methods:**

Thirty-seven eyes with NIU including Behçet disease (BD), sarcoidosis, ankylosing spondylitis (AS), juvenile idiopathic arthritis and idiopathic arthritis, 38 eyes of non-uveitic (NU) patients including BD, AS and rheumatoid arthritis, and 40 healthy control eyes were included. ADA was used for anti-TNF-naive adult (80 mg) or paediatric (40 mg) patients with refractory NIU, then 40 mg every 2-week (20 mg in children<30 kg) with controls at weeks 1, 4, 12, and 24. Images were used to measure central, nasal, and temporal ChT, and the luminal area (LA), stromal area, and total choroidal area (TCA) were analysed using enhanced-depth imaging optical coherence tomography (EDI-OCT) by ImageJ software. The CVI was then calculated as the ratio of LA to TCA.

**Results:**

Mean ages were similar between the groups. Mean (SE) subfoveal ChT measurements for each location were also similar (for each, *p* > 0.05). However, calculated CVI values in eyes with NIU (0.63 ± 0.007) were significantly (*p* < 0.001) lower than NU eyes (0.66 ± 0.006) and controls (0.70 ± 0.007) (*p* < 0.001). Moreover, CVI was significantly lower in NU eyes compared to controls (*p* < 0.001). There were no significant CVI changes between the consecutive visits after ADA therapy in eyes with NIU (for each, *p* > 0.05).

**Conclusions:**

Decreased CVI in NIU and NU eyes indicates that systemic inflammation affects the choroidal vasculature and perfusion both in the presence and absence of ocular involvement. Although CVI may be used as a possible novel tool in monitoring ocular involvement and progression of NIU, CVI does not seem to be a biomarker for treatment monitoring in NIU.

## Introduction

Uveitis is the leading cause of blindness in the developed world, accounting for 10–15% of all blindness cases. [1–4] Non-infectious uveitis (NIU) can be caused by various systemic or ocular autoimmune rheumatological disorders [5] including Behçet disease (BD), HLA-B27-associated uveitis, juvenile idiopathic arthritis (JIA), ankylosing spondylitis (AS), rheumatoid arthritis (RA), and Vogt-Koyanagi-Harada (VKH) disease. However, patients with systemic rheumatological disorders without ocular involvement (non-uveitic=NU) may also be encountered.

There are several immunosuppressive drugs used in the treatment of NIU. [6] Although corticosteroids are commonly preferred, immunomodulatory steroid-sparing agents such as alkylating agents, antimetabolites, and calcineurin inhibitors are necessary in cases with panuveitis. [7] Tumour necrosis factor-alpha (TNF-α) is a pro-inflammatory cytokine that has been implicated in the pathogenesis of NIU, [8] and anti-TNF-α drugs such as infliximab, [9] daclizumab, and adalimumab (ADA) [10] have been used to selectively inhibit this cytokine as a novel modern therapy in refractory cases. [7, 11]

It is important to monitor disease activation, complications, and the effects of systemic treatment in patients with uveitis. Optical coherence tomography (OCT) is used to monitor retinal complications and provides information on macular thickness (MT). Although various studies have investigated choroidal thickness (ChT) as an indicator of disease activity, its measurement does not objectively represent the entire choroidal vascular structure, and ChT is influenced by many factors such as age, sex, axial length, refractive error, and intraocular pressure (IOP). [12] Enhanced-depth imaging OCT (EDI-OCT), in turn, is a non-invasive technique that provides deeper choroidal accessibility and visibility. [13] The choroid’s vascular-luminal area (LA), interstitial-stromal area (SA), and subfoveal-total choroidal area (TCA) can be measured separately using EDI-OCT. The novel choroidal vascularity index (CVI) is then calculated as the ratio of LA/TCA that demonstrates the % proportion of vascularity within the choroidal tissue. Therefore, CVI is thought to be more stable and less variable than ChT in the presence of abnormalities in choroidal microvascular structure, which may be used as a more specific, quantitative, and reliable biomarker in numerous retinal and choroidal diseases. [14]

No studies investigated the effect of ADA treatment on the choroidal microvascular structure in patients with NIU. Therefore, we aimed to perform a comparison of CVI between eyes with NIU, NU patients, and control subjects and to investigate the effect of ADA therapy on ChT and CVI in NIU.

## Materials and methods

All procedures involving human subjects were in accordance with the tenets of the Helsinki Declaration in 1964. Informed consent was obtained from all participants and the study protocol was approved by the Ethical Committee of Erciyes University Medical Faculty (Approval No: 2022/339).

### Study design and participants

This retrospective, cross-sectional, and comparative study evaluated **(1)** eyes with NIU, **(2)** patients with non-ocular rheumatological disorders (NU patients) who were referred by rheumatology and/or dermatology clinics to our tertiary clinic for routine uveitis screening, and **(3)** age- and gender-matched healthy control subjects with no other ocular/systemic diseases. Medical records of patients were reviewed at the Uvea-Behçet Unit of Erciyes University Medical Faculty between 2021 and 2022. Demographic information (age, sex), disease duration, and diagnosis were recorded. Six-month longitudinal data of patients using ADA for uveitis were collected. Eyes with NIU were evaluated at baseline, week 1, week 4, week 12, and week 24. The diagnosis of BD was performed on the International Criteria for Behçet’s Disease with a total score of ≥4 points. [15]

### Exclusion-inclusion criteria and examinations

Exclusion criteria for all participants were as follows: diabetes mellitus, hypertension, pregnancy, glaucoma, history of ocular surgery/trauma/laser intervention, any types of optic neuropathies, refractive errors (>±3 D), axial lengths over 24 mm, and low-quality OCT images. Anti-TNF-naïve patients with NIU were refractory to the combination of corticosteroids (methylprednisolone, 1 mg/kg/day) plus one of the following immunosuppressants such as methotrexate (MTX), cyclosporine, azathioprine (AZT), or interferon for at least 12 weeks or longer. NU patients (non-ocular) were excluded if they had past history or findings of a previous uveitis attack such as anterior chamber (AC) or vitreal cells, keratic precipitates, synechia formation, iris pigments on the lens capsule, optic disc pallor/atrophy/neovascularization, and retinal vascular sheathing.

### ADA therapy protocol

Subcutaneous injection of ADA was given as a maintenance dose of 40 mg every two weeks after a loading dose of 80 mg in adults and children weighed ≥30 kg. If the child weighed <30 kg, it was given as a maintenance dose of 20 mg every two weeks after a loading dose of 40 mg. Malignancies and systemic infectious diseases were excluded before starting ADA treatment, which were checked at three-month intervals using safety follow-up forms. Systemic corticosteroids, if present, were stopped within 2 to 4 weeks from the initiation of ADA injections. Immunosuppressives were gradually reduced at 4 to 6 weeks, but not completely stopped. Topical steroid treatment, if present, was continued.

### Ocular examination

All patients and control individuals underwent a detailed biomicroscopic evaluation of the anterior segment and retina using a + 90D lens. OCT scans were performed by an experienced technician for all subjects. Best corrected visual acuity (BCVA) was expressed as the logarithm of the Minimum Angle of Resolution (logMAR) as measured with a Snellen chart (20 ft.). Goldmann applanation tonometry was used for IOP measurements. The scoring system recommended by the Standardisation of Uveitis Nomenclature Working Group and adapted National Eye Institute criteria under the regimen was used for anterior chamber cell (ACC) grade and vitritis grade. [16] Therefore, ‘active’ eyes had at least one active inflammatory chorioretinal lesion, ACC grade of ≥1 + , or vitreous haze grade of ≥1 + . ‘Inactive’ eyes had no inflammatory chorioretinal lesions and an ACC grade and vitreous haze grade ≤0.5 +. Central MT and ChT measurements were recorded and CVI was calculated. The anatomical location of ocular involvement was noted in patients with NIU and HLA analyses were performed. Concomitant immunosuppressive drugs such as MTX, AZT, and/or corticosteroid therapy doses were recorded, if present.

The spectral domain (SD)-OCT (Spectralis HRA + OCT, Heidelberg Engineering GmbH, Heidelberg, Germany) was applied to all participants. When needed, standard automated perimetry analysis (Humphrey Visual Field Analyser 750i; Carl Zeiss Meditec Inc., Dublin, CA, USA), fundus autofluorescence (Spectralis HRA + OCT, Heidelberg Engineering GmbH, Heidelberg, Germany), and fluorescein angiography (KOVA imaging systems, Japan) were performed in patients with a suspected diagnosis to exclude ocular involvement.

### OCT scanning protocol and image processing

**Fig. 1:**
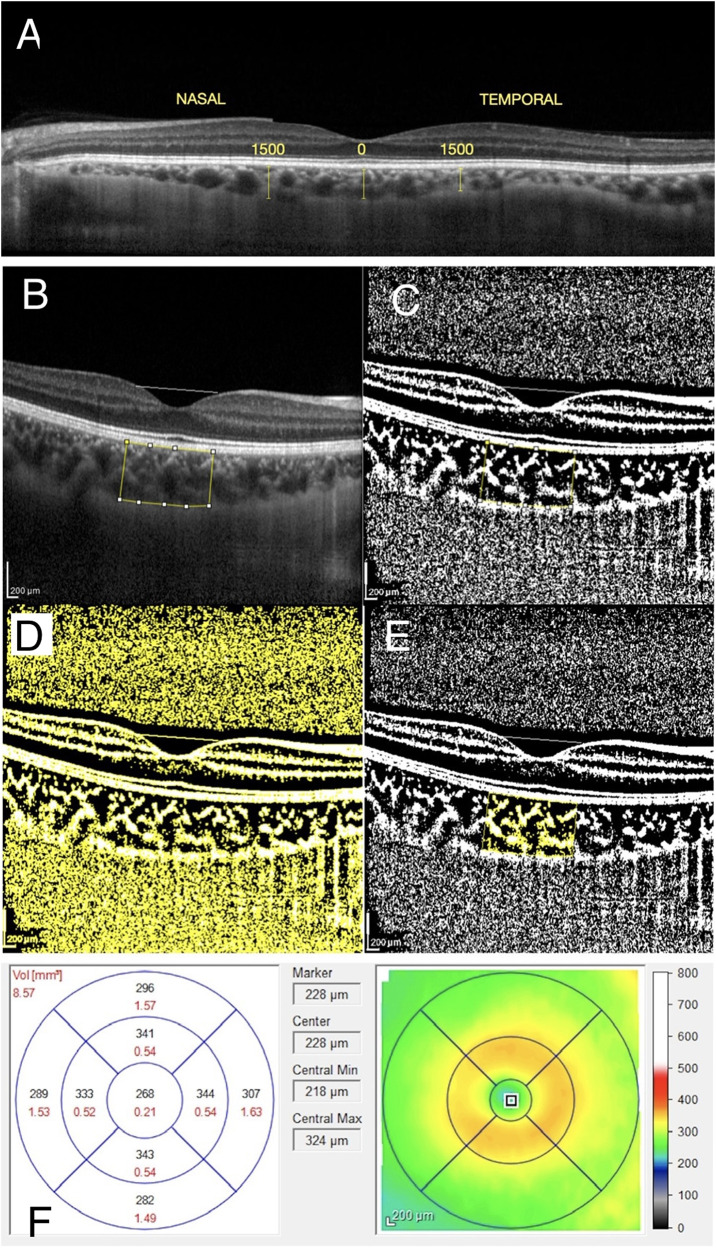
Illustrations of the submacular choroidal thickness measurements, choroidal vascularity index analysis, and macular thickness measurements using enhanced-depth imaging optical coherence tomography. **A** Submacular choroidal thickness measurements: Choroidal thickness was measured at 3 locations: subfoveal region, 1500 μm nasal (N1500), and 1500 μm temporal (T1500) to the fovea. The measurement was performed manually from the outer part of the retinal pigment epithelium to the inner scleral border. **B**, **C**, **D**, **E** A step-by-step explanation of image binarization using ImageJ software for choroidal vascularity index analysis: **B**﻿ A reference line was drawn parallel to the retinal pigment epithelium (fovea-centred, 1500 μm total length). Taking into account the reference line, the subfoveal total choroidal area between the retinal pigment epithelium and the choroidal-scleral junction was selected using the polygon tool. **C** The image was converted to an 8-bit image and autolocal thresholding was applied. **D** Niblack was selected to obtain a clear segmentation of black and white areas of the choroid. **E** The binarized image was converted back to an RGB image. **F** Retinal thickness map of the macula with EETDRS thickness analysis in micrometres: The macula is divided into 9 regions; a central circle of 500 μm radius, and inner and outer rings, each divided into four quadrants. An average central retinal thickness is calculated in these regions.

OCT imaging of the macular region was performed with a Spectralis domain OCT (Heidelberg Engineering, GmbH, Heidelberg, Germany) with software version 6.3.3.0, using the EDI-OCT protocol with 20° × 20° for all participants. Central MT was measured automatically by the Spectralis software. ChT was measured at three different points for each eye in the subfoveal region, 1500 μm nasal (N1500), and 1500 μm temporal (T1500) to the fovea. The measurement was made manually with the calliper tool from the outer part of the retinal pigment epithelium (RPE) to the inner scleral border. [17] ChT measurements are shown in Fig. 1A.

For CVI, EDI mode images were analysed using an open source ImageJ software (version 1.53i, National Institutes of Health, USA) as described by Agrawal et al. [18] The image was converted to an 8-bit type, and the automatic local thresholding tool was used for binarization (Niblack’s method). A reference line was drawn parallel to the RPE centring on the fovea (1500 μm total length). The polygon tool was then used to select the subfoveal circumscribed TCA between the outer surface of the RPE and the choroidal-scleral interface. The first image was added to the region of interest (ROI) manager tool. The image was then reconverted to a red-green-blue colour type, the threshold colour was selected as white, and the image was added to the ROI manager again. White pixels corresponded to the interstitial SA and dark/black pixels represent the vessels’ LA (vascularity area). Both images were merged into a third image in the ROI manager and were added to the ROI manager tool again. The software automatically calculated the images. The calculation of the first image showed the TCA and the measurement of the last image showed the SA. The SA was subtracted from the TCA to obtain the LA. The CVI value (%) was estimated as the ratio of LA to TCA. To prevent diurnal variation, all the EDI-OCT images were obtained by the same technician between 9 and 11 AM. The measurement of CVI is shown in Fig. 1B, C, D, E.

The distance from the internal limiting membrane (ILM) to the RPE was defined as the central MT. The macula was divided into nine regions: a central circle of 500 μm radius, an inner ring, and an outer ring, each divided into four quadrants. [19] An average central MT was calculated in these regions. MT measurements are shown in Fig. 1F. All OCT images and analyses were repeated at baseline, week 1, week 4, week 12, and week 24 for patients with NIU. ChT measurements and CVI calculations were performed separately by two ophthalmologists (EEA, ZA) blind to each other’s readings and measurements were averaged.

### Statistical analysis

Statistical analysis was performed using SPSS version 22 (SPSS Inc., IBM, Chicago, IL, USA). The Shapiro-Wilk test for normality and the Levene test for homogeneity of variance were performed on the data set. Descriptive nominal data were expressed as percentages. The Pearson *chi*-squared test was used for nominal data. One-way ANOVA was used for normally distributed data and the Kruskal-Wallis test was used for non-normally distributed data. A linear mixed-effect model was used to compare continuous parameters longitudinally and cross-sectionally, adjusting for pairwise correlations. Data are expressed as mean (standard error=SE) or median (quartiles 25%–75%), depending on the distribution. *P* < 0.05 were considered significant. Bonferroni adjusted *p* values were reported.

## Results

### Demographic characteristics of patients

A total of 37 eyes of 19 patients (7 male, 12 female; 11 adults, 8 children) with unilateral (*n* = 1) or bilateral (*n* = 18) NIU with the mean age of 30.3 ± 4.2 years (range, 7–65) and 38 eyes of 19 NU patients (2 male, 17 female) with the mean age of 33.2 ± 3.9 years (range, 10–64) were included in the study. Forty eyes of 20 subjects (8 male, 12 female) with a mean age of 34.2 ± 1.7 years (range, 26–50) were included as healthy controls in the study. There was no statistically significant difference in the distribution of mean age or sex between the three groups (*p* > 0.05). The demographic data are shown in Table 1.

**Table 1:** Baseline characteristics of patients with non-infectious uveitis, non-uveitic rheumatological patients, and healthy controls.

Variables	Uveitis (Mean ± SE)	Non-Uveitis (Mean ± SE)	Control (Mean ± SE)
Age (years)	30.3 ± 4.2	33.2 ± 3.9	34.2 ± 1.7
Gender (male/female)	7/12	2/17	8/12
BCVA (logMAR)	0.42 ± 0.05	0.01 ± 0.05	0.00 ± 0.05
IOP (mm/Hg)	13.8 ± 0.4	15.8 ± 0.4	14.5 ± 0.4
Azathioprine (mg/day)	100.0 ± 20.4	105.0 ± 12.2	-
Steroid (mg/day)	58.6 ± 14.1	-	-
Colchicine (mg/day)	-	1.5 ± 0.1	-
Methotrexate (mg/day)	14.5 ± 1.3	15.0 ± 2.0	-

*BCVA* best-corrected visual acuity, *IOP* intraocular pressure.

In the NIU group, the mean disease duration before ADA therapy was 56.0 ± 19.9 months. NIU patients were diagnosed with BD in 6 (32%), sarcoidosis in 1 (5%), AS in 1 (5%), JIA in 1 (5%), and idiopathic arthritis in 10 (53%) patients. Two patients (11%) had anterior uveitis and vitritis, 7 (37%) had intermediate uveitis, and 9 (47%) had posterior uveitis. The remaining inactive one patient (5%) demonstrated frequent relapses with the conventional regimen. Thirty-five of 37 eyes had active uveitis and the remaining 2 eyes had inactive uveitis (with frequent relapse) before ADA therapy. Before treatment, 1 patient (5%) was on pulse steroid therapy (1 g/day), 3 patients (16%) on oral steroid therapy (1 mg/kg/day), 6 patients (32%) on MTX, and 4 (21%) on AZT therapy. The mean daily steroid dose was 58.6 ± 14.1 mg. MTX and AZT doses were 14.5 ± 1.3 mg and 100.0 ± 20.4 mg, respectively. Five patients (26%) were HLA-B27 positive and 4 patients (21%) were HLA-B51 positive.

In NU patients group, the diagnosis was BD in 10 patients (52%), AS in 5 (26%), and RA in 4 (21%) patients. Eight patients (42%) were on colchicine therapy, 4 (21%) on MTX, and 5 (26%) on AZT therapy for their systemic non-ocular disease. The mean daily dose of colchicine was 1.5 ± 0.1 mg. The daily doses of MTX and AZT were 15.0 ± 2.0 mg and 105.0 ± 12.2 mg, respectively. None of the patients in this group received ADA.

### Longitudinal comparison of patients with NIU

The mean BCVA (logMAR) was 0.42 ± 0.10 at baseline. After ADA initiation, there was a significant increase in BCVA at week 12 (*p* = 0.020) and week 24 (*p* = 0.025) compared to baseline. Longitudinal follow-up comparisons are shown in Table 2. The mean BCVA was 0 logMAR in both NU patients and controls. ACC grade was 0.9 ± 0.1 and vitritis grade was 0.84 ± 0.08 at baseline. The outcomes of ADA treatment were analysed based on the last visit. Complete, rapid, and sustained control of both anterior and posterior uveitis was observed in all eyes without relapses during the follow-up. Both ACC and vitritis cell grades started to decrease within the first week and improvement was observed until the last visit at 24 weeks (for each, *p* ˂ 0.001). Similarly, the mean central MT was 312.0 µm at baseline, which decreased to 275.8 µm at the last visit (*p* = 0.033). In addition, central ChT significantly decreased (*p* = 0.011) at the last visit (284.4 ± 8.9 µm) compared to baseline (303.6 ± 8.4 µm). There was a significant reduction (*p* = 0.010) in nasal ChT at week 24 (238.6 ± 9.8 µm) compared to baseline (254.9 ± 9.3 µm). However, no significant difference was observed for temporal ChT (*p* > 0.05). Similarly, CVI in NIU patients did not change during the follow-up period (for each, *p* > 0.05). No ocular complications emerged during the management period. Baseline ocular measurements and longitudinal follow-up comparisons for BCVA, CVI, intraocular inflammation grade, and choroidal-retinal measurements are shown in Table 2.

**Table 2:** Longitudinal analysis of patients with non-infectious uveitis treated with adalimumab.

Variables (*N* = 37)	Baseline^a^ (Mean ± SE)	1th Week^b^ (Mean ± SE)	4th Week^c^ (Mean ± SE)	12th Week^d^ (Mean ± SE)	24th Week^e^ (Mean ± SE)	*p*
CVI	0.63 ± 0.006	0.63 ± 0.006	0.64 ± 0.006	0.64 ± 0.006	0.64 ± 0.007	For each, *p* > 0.05
Nasal ChT (µm)	254.9 ± 9.3	261.5 ± 9.5	249.7 ± 9.4	264.7 ± 9.3	238.6 ± 9.8	***p***^a-e^ = **0.010,** ***p***^b-e^ = **0.021,** ***p***^d-e^ = **0.004**
Central ChT (µm)	303.6 ± 8.4	298.1 ± 8.5	291.5 ± 8.4	294.6 ± 8.4	284.4 ± 8.9	***p***^a-e^ = **0.011**
Temporal ChT (µm)	271.9 ± 8.7	263.1 ± 8.9	259.3 ± 8.7	262.8 ± 8.7	263.9 ± 9.3	For each, *p* > 0.05
Nasal MT (µm)	369.2 ± 11.5	364.4 ± 11.6	358.9 ± 11.5	362.5 ± 11.5	354.7 ± 11.9	For each, *p* > 0.05
Central MT (µm)	312.0 ± 13.9	293.9 ± 14.2	291.8 ± 13.9	288.2 ± 13.9	275.8 ± 14.9	***p***^a-e^ = **0.033**
Temporal MT (µm)	360.7 ± 11.2	351.3 ± 11.4	351.6 ± 11.2	348.6 ± 11.2	338.2 ± 11.9	*p* > 0.05
BCVA (LogMAR)	0.42 ± 0.1	0.35 ± 0.1	0.33 ± 0.1	0.27 ± 0.1	0.26 ± 0.1	***p***^a-e^ = **0.025,** ***p***^a-d^ = **0.020**
ACC Grade	0.9 ± 0.1	0.5 ± 0.1	0.3 ± 0.1	0.1 ± 0.1	0.1 ± 0.1	***p***^a-b^ = **0.032,** ***p***^b-d^ = **0.017,** ***p***^a-c,d,e^ < **0.001**
Vitritis Grade	0.84 ± 0.08	0.15 ± 0.08	0.05 ± 0.08	0.11 ± 0.08	0.12 ± 0.09	***p***^a-b,c,d,e^ < **0.001**

*ACC* anterior chamber cell, *BCVA* best-corrected visual acuity, *ChT* choroidal thickness, *CVI* choroidal vascularity index, *MT* macular thickness. P values in bold indicate statistically significant results.

### Subgroup longitudinal analysis for Behçet patients and idiopathic uveitis

Subgroup analysis demonstrated that the CVI did not change during the follow-up period both in Behçet (Table 3) and idiopathic patients (Supplemental Data 1) (for each, *p* > 0.05). In idiopathic patients, central ChT significantly (*p* = 0.022) decreased at the last visit (276.3 ± 11.6 µm) compared to baseline (305.8 ± 11.1 µm) (Supplemental Data 1). Similarly, significantly decreased nasal (*p* = 0.009) and central (*p* = 0.02) MTs were found when compared with baseline values. However, no significant differences were observed for all other comparisons (*p* > 0.05).

**Table 3:** Longitudinal analysis of Behçet patients treated with adalimumab.

Variables (*N* = *12*)	Baseline (Mean ± SE)	1th Week (Mean ± SE)	4th Week (Mean ± SE)	12th Week (Mean ± SE)	24th Week (Mean ± SE)	*p*
CVI	0.63 ± 0.009	0.63 ± 0.011	0.63 ± 0.009	0.61 ± 0.009	0.62 ± 0.011	For each*, p* > 0.05
Nasal ChT (µm)	263.5 ± 14.8	283.5 ± 15.8	257.2 ± 15	281.6 ± 14.8	256.6 ± 15.8	For each*, p* > 0.05
Central ChT (µm)	319.8 ± 15.5	319.8 ± 17.1	303.8 ± 15.8	315.3 ± 15.5	306.7 ± 17.1	For each, *p* > 0.05
Temporal ChT (µm)	267.8 ± 12.5	265.2 ± 15.4	266.4 ± 12.8	261.3 ± 12.5	272.4 ± 14.4	For each, *p* > 0.05
Nasal MT (µm)	318.1 ± 23.2	318.2 ± 23.5	320.5 ± 23.2	322.6 ± 23.2	336.2 ± 23.5	For each*, p* > 0.05
Central MT (µm)	234.7 ± 21.4	231.6 ± 21.9	235.4 ± 21.5	239.2 ± 21.4	251.3 ± 21.9	For each*, p* > 0.05
Temporal MT (µm)	309.7 ± 22.4	304.0 ± 22.8	311.7 ± 22.5	310.9 ± 22.4	316.6 ± 22.8	For each, *p* > 0.05

*ChT* choroidal thickness, *CVI* choroidal vascularity index, *MT* macular thickness.

### Cross-sectional comparison between groups

**Fig. 2:**
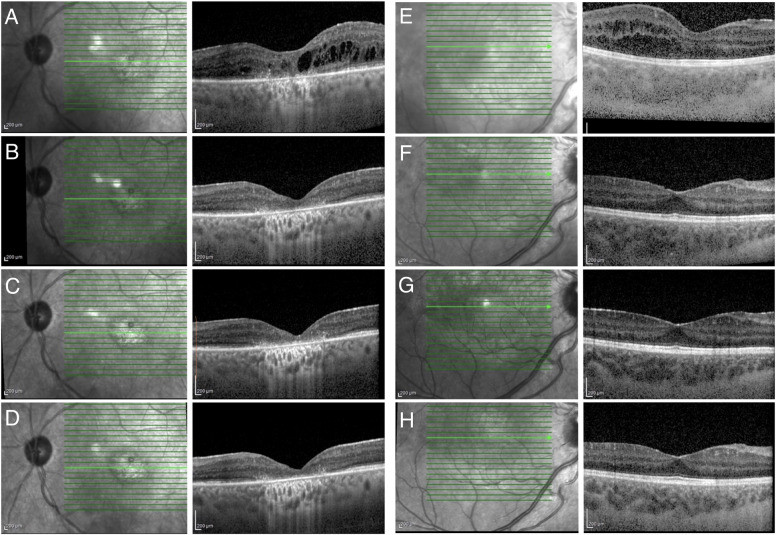
Horizontal spectral-domain optic-coherence tomography images of the macula regions for 2 patients. **A** The left eye of a 61-year-old woman with ankylosing spondylitis who had non-infectious uveitis showed the presence of cystoid oedema in the central macula before adalimumab treatment. **B** 1 week after adalimumab treatment demonstrates a fast resolution of cystoid macular oedema. **C**, **D** Sustained effectiveness of adalimumab treatment at 4 and 24 weeks, respectively. **E** The right eye of a 7-year-old boy who had idiopathic non-infectious uveitis demonstrated the presence of cystoid macular oedema before adalimumab initiation. **F** Resolution of oedema at 1 week following adalimumab therapy, which remained stable at 4 weeks (**G**) and 24 weeks control (**H**).

Taken together, there was no significant difference in central, nasal, and temporal ChT between the groups (for each, *p* = 1.000). However, CVI was significantly lower in eyes with NIU (0.63 ± 0.007) compared to NU patients (0.66 ± 0.006, *p* = 0.004) and healthy control subjects (0.70 ± 0.007, *p* < 0.001). Central MT was significantly higher in eyes with NIU (312.0 ± 9.8 µm) compared to NU patients (265.6 ± 9.6 µm, *p* = 0.003) and controls (266.0 ± 9.4 µm, *p* = 0.003). Similar results were observed for both nasal and temporal MT. Cross-sectional comparisons between the groups are shown in Supplemental Data 2. Baseline MT status and the effectiveness of ADA treatment during follow- up at weeks 1, 4, and 24 are shown in Fig. 2A, B, C, D for an adult patient with NIU (AS) and in Fig. 2E, F, G, H for a child with idiopathic NIU.

## Discussion

The choroid is a vascular and highly perfused tissue that is affected by many multisystemic autoimmune and inflammatory diseases with or without ophthalmologic manifestations. [20] Therefore, changes in this tissue could be used as an ocular indicator of many systemic autoimmune disorders. In the present study, we investigated retinal and choroidal changes in eyes with NIU and NU patients and evaluated the longitudinal effect of ADA treatment on visual acuity, intraocular inflammation, ChT, MT, and CVI compared to healthy controls. Our results demonstrated that MT was increased in eyes with NIU compared to NU patients and controls. ADA treatment improved VA and decreased both anterior and posterior segment inflammation, which started to improve in the first week. In addition, ADA therapy significantly reduced central and nasal ChT at consecutive visits and central MT at week 24. On the other hand, CVI values were found to be decreased in eyes with NIU compared to NU patients and controls. In addition, NU patients also demonstrated decreased CVI values compared to controls.

NIU is an inflammatory status that may affect the AC, vitreous, retina, and choroid, requiring systemic immunosuppressive agents. In a recent meta-analysis, we have reported that ADA was shown to improve vision in BD by reducing inflammation, macular oedema, and retinal vasculitis [9] and that recalcitrant posterior segment involvement in children with NIU is effectively treated with ADA. [21] However, the monitoring tools for subclinical or clinical ocular involvement in NIU are limited. If we cannot find a reliable biomarker to diagnose early ocular involvement and monitor disease progression, patients with NIU may be undiagnosed in time and under or over-treated.

Numerous imaging tools are used to diagnose and monitor disease progression in retino-choroidal inflammatory disorders such as fundus fluorescein angiography for the detection of retinal vasculitis and indocyanine green angiography for choroidal pathology and vascularity. [22] However, both methods are relatively invasive and are not practically used for routine follow-up. As a result, the non-invasive EDI-OCT allows quantitative structural measurements of the choroidal vasculature in ocular inflammation. Therefore, calculating the CVI% (LA/TCA) provides a more stable and repeatable biomarker that shows the proportion of vascularity within the choroid. [23]

Since diurnal variation, refractive error, gender, and age affect the status of the ChT, it may not be a diagnostic method in NIU and baseline ChT was similar between eyes with NIU, NU patients, and controls. All these findings for ChT indicate that a novel alternative biomarker is of utmost importance to diagnose and follow up patients with active and inactive NIU. In addition to the ChT measurements, therefore, we included quantitative segmentation of the choroid into vascular LA and interstitial SA using EDI-OCT images, which may be used as a reproducible and repeatable diagnostic tool for rheumatological disorders with or without ocular involvement. [22, 23] Indeed, we do not presently know which structures within the choroid exhibit changes in patients with posterior segment inflammation, especially in the absence of ChT changes. In the present research, the analysed CVI values in NIU and NU patients were significantly lower than that in control subjects. In addition, the CVI value in NIU was also lower than that in NU patients. Such results indicate that systemic rheumatological vasculitic disorders with or without ocular inflammation affect not only the retina but also the choroid. In other words, % change in CVI informs us on the proportion of vascularity and, therefore, indirectly about the choroidal perfusion and ischemia, indicating decreased vascular LA with or without interstitial SA expansion as a result of inflammatory response in the inner stroma near the retina, which is not associated with choroidal thickening. Indeed, because we found unchanged ChT in NIU and NU patients compared to controls, calculating the CVI value may be a more robust optical biomarker than ChT for detecting subclinical or clinical choroidal changes in non-ocular or ocular rheumatological disorders, respectively. It may be used as a novel tool to monitor ocular involvement and its progression in such systemic inflammatory disorders, since CVI was also significantly different between the NIU and NU patients.

CVI is said to be used as a novel biological indicator in disease progression and follow-up in some inflammatory disorders. [18] However, conflicting results have recently been reported for CVI values in HLA-B27-associated uveitis, JIA, and Fuchs uveitis syndrome (FUS). Increased CVI values have been reported in eyes with quiescent VKH syndrome [24] and acute HLA-B27-related anterior uveitis without posterior segment involvement, suggesting choroidal vascular engorgement. [25] On the other hand, CVI was found to be decreased when granulomatous anterior uveitis appeared in patients with VKH [24, 26] and intermediate uveitis. [13] Similarly, three recent studies have reported decreased CVI values in eyes with both JIA-associated uveitis and FUS compared to non-uveitic fellow eyes and healthy control individuals. [26–28] The authors proposed that continuous inflammation results in inflammation-mediated thinning in the vascular area of the choroid. Likewise, Agrawal et al. [18] have demonstrated a decrease in CVI at 3 months follow-up in patients with panuveitis. Moreover, Şimşek et al. [29] showed that CVI was significantly lower in non-uveitic patients with BD compared to healthy controls. Therefore, our findings were consistent with these current articles and reported significantly decreased CVI values in eyes with NIU and NU patients compared to controls. Therefore, changes in choroidal OCT parameters for such patients in the absence of uveitis may indicate subclinical choroidal changes during systemic inflammation.

Available evidence suggests that TNF-α levels are increased in uveitis [8, 9] and effective immunosuppression can be obtained with anti-TNF-α agents in NIU including BD. [6, 30, 31] We have recently reported that infliximab [10] and ADA [21] is a safe and effective treatment to suppress inflammation in paediatric patients with NIU including BD and JIA with no serious adverse effects and that steroids could be stopped after anti-TNF therapy. The present study supports this steroid-sparing effect, as all steroids were stopped in NIU patients after the start of ADA treatment. However, the present research has demonstrated that ADA treatment does not seem to affect the calculated CVI values at least in the weeks of follow-up time and, therefore, it may not be used as a biomarker for treatment monitoring in patients with NIU. Indeed, CVI changes with treatment response are also controversial in the literature. Patients with acute VKH syndrome showed a significant reduction in CVI after corticosteroid treatment [32, 33] whereas CVI values were found to be increased with the resolution of inflammation in patients with VKH and intermediate uveitis [13, 24] In the present study, the fact that we did not find a significant change in CVI after ADA treatment could be a sign of an ongoing systemic inflammatory process. Although 24 weeks of ADA therapy reduced inflammation in the anterior and posterior segments and decreased MT, it may be concluded that this was not enough time to change the choroidal vascular or stromal structures. The conflicting results of longitudinal analysis of CVI in the literature may be due to heterogeneity in the number and disease activity between patients, treatment modality, differences in follow-up duration and treatment, and the inflammation status.

MT is an indicator of ocular inflammation in systemic inflammatory diseases and an association has been demonstrated between central foveal thickness and anterior segment inflammation in patients with spondyloarthropathy, [34] demonstrating subclinical posterior segment involvement as a result of increased vascular permeability. In a study investigating central and perifoveal retinal thickness in intermediate uveitis, Gehl et al. [35] reported that retinal thickness was significantly higher compared to healthy controls. Therefore, both the literature and our results show that retinal thickening measured by OCT is a useful clinical parameter not only to assess the degree of inflammation but also to monitor response to treatment instead of CVI values. [35–37]

Our study has several limitations. First, the sample size was small and the diagnosis of the patients and treatment were not homogenous. Therefore, further studies can be performed with one type of diagnosis (such as patients with BD). Second, eyes with dense vitreous inflammation were not included in the study due to poor image quality, and only high-resolution EDI-OCT images were included. Third, only the central submacular 1500 μm of the choroid was calculated, and a larger choroidal area may reveal global information. Finally, the CVI was not correlated with some alternative biomarkers of ocular inflammation such as ACC grade and vitritis grade, which need further cross-sectional, longitudinal, and comparative research for potential future applications of CVI in such patients with or without ocular involvement.

In conclusion, ADA treatment is effective in NIU, reduces active intraocular inflammation parameters with the resolution of macular oedema, and improves final VA. Because ChT is affected by numerous physiological factors, the calculated CVI value may quantitatively determine the choroidal vascularity, overcoming the limitations of ChT alone, and may be used as a new, repeatable, and robust optical indicator not only to evaluate choroidal abnormalities in NIU patients with sight-threatening posterior or panuveitis but to monitor their chronicity. In addition, the choroidal vascular structure may be affected prior to retinal changes in patients with systemic rheumatological disorders. However, the effectiveness of management during the disease course may not be guided by this new index.

## Summary

### **What was known before**

- Adalimumab is a well-known anti-TNF agent for the treatment of non-infectious uveitis and no studies investigated the effect of adalimumab treatment on the choroidal microvascular structure in patients with non-infectious uveitis.

### **What this study adds**

- We evaluated choroidal microvasculature in adult and paediatric patients with ocular and non-ocular rheumatological disorders assessed with enhanced-depth imaging optical coherence tomography-based binarization, and evaluated the effect of adalimumab therapy on these parameters.
- A decreased choroidal vascularity index was detected under the central macular region in eyes with both non-infectious uveitis and non-uveitic (non-ocular) rheumatological patients.
- Subclinical quantitative changes in choroidal microcirculation develop prior to retinal changes in non-ocular patients, which may indicate subclinical ocular inflammation.
- This novel, non-invasive, and repeatable optical indicator may quantitatively determine and monitor the changes in choroidal microvascular structure, overcoming the limitations of choroidal thickness alone.
- The effectiveness of adalimumab treatment during the disease course, however, may not be guided by this new index.

### Supplementary information

Supplemental Data 1

Supplemental Data 2

## Data Availability

All data generated or analysed during this study are available on request.
