# Supplementary material for: Effect of adalimumab on choroidal thickness and choroidal vascularity index in eyes with non-infectious uveitis using enhanced-depth imaging optical coherence tomography
Source: Eye (Lond). 2024 Feb 20;38(9):1633–41. doi: 10.1038/s41433-024-02975-9 (PMC11156944; doi:10.1038/s41433-024-02975-9)
Supplement: Supplementary file 1 — Supplemental Data 1 [file 41433_2024_2975_MOESM1_ESM.docx]

| **Supplemetal Data 1.** Longitudinal analysis of idiopathic patients treated with adalimumab. | | | | | | |
| --- | --- | --- | --- | --- | --- | --- |
| **Variables** ( *N=12*) | **Baseline^a^**  (Mean±SE) | **1^th^ Week^b^**  (Mean±SE) | **4^th^ Week^c^**  (Mean±SE) | **12^th^ Week^d^**  (Mean±SE) | **24^th^ Week^e^**  (Mean±SE) | ***p*** |
| **CVI** | 0.64±0.008 | 0.65±0.008 | 0.66±0.008 | 0.66±0.008 | 0.66±0.008 | For each, *p*>0.05 |
| **Nasal ChT** (µm) | 261.3±13.3 | 263.9±13.3 | 256.3±13.3 | 265.7±13.3 | 234.5±13.9 | For each, *p*>0.05 |
| **Central ChT** (µm) | 305.8±11.1 | 298.4±11.1 | 296.0±11.1 | 290.3±11.1 | 276.3±11.6 | ***p^a-e^*=0.022** |
| **Temporal ChT** (µm) | 293.9±13.4 | 270.9±13.4 | 266.3±13.4 | 277.0±13.4 | 269.1±14.1 | For each, *p*>0.05 |
| **Nasal MT** (µm) | 408.1±12.6 | 399.5±12.6 | 390.4±12.6 | 391.7±12.6 | 372.6±13.2 | ***p^a-e^*=0.009** |
| **Central MT** (µm) | 359.5±18.5 | 336.0±18.5 | 323.7±18.5 | 310.6±18.5 | 299.2±19.7 | ***p^a-e^*=0.02** |
| **Temporal MT** (µm) | 391.0±12.9 | 386.3±12.9 | 380.5±12.9 | 373.2±12.9 | 356.4±14.1 | For each, *p*>0.05 |
| ChT=choroidal thickness; CVI=choroidal vascularity index; MT=macular thickness. | | | | | | |
