# Supplementary material for: Effect of adalimumab on choroidal thickness and choroidal vascularity index in eyes with non-infectious uveitis using enhanced-depth imaging optical coherence tomography
Source: Eye (Lond). 2024 Feb 20;38(9):1633–41. doi: 10.1038/s41433-024-02975-9 (PMC11156944; doi:10.1038/s41433-024-02975-9)
Supplement: Supplementary file 2 — Supplemental Data 2 [file 41433_2024_2975_MOESM2_ESM.docx]

| **Supplemetal Data 2.** Cross-sectional analysis between the groups. | | | | | | | |
| --- | --- | --- | --- | --- | --- | --- | --- |
| **Variables** | **Uveitis**^a^  ***N***=37  (Mean±SD) | **Non-Uveitis**^b^  ***N***=38  (Mean±SD) | **Control**^c^  ***N***=40  (Mean±SD) | ***p*** | ***p****^a-c^* | ***p****^b-c^* | ***p****^a-b^* |
| **CVI** | 0.63±0.007 | 0.66±0.006 | 0.70±0.007 | **<0.001** | **<0.001** | **<0.001** | **0.004** |
| **Nasal ChT** (µm) | 254.8±9.1 | 242.0±9.0 | 249.2±8.7 | 1.000 | 1.000 | 1.000 | 0.960 |
| **Central ChT** (µm) | 303.6±8.4 | 290.5±8.8 | 297.1±8.6 | 1.000 | 1.000 | 1.000 | 0.906 |
| **Temporal ChT** (µm) | 271.8±8.9 | 250.4±8.8 | 268.6± 8.6 | 1.000 | 1.000 | 0.436 | 0.279 |
| **Nasal MT** (µm) | 369.2±7.6 | 341.2±7.5 | 343.4±7.3 | **<0.001** | **0.050** | 1.000 | **0.031** |
| **Central MT** (µm) | 312.0±9.8 | 265.6±9.6 | 266.0±9.4 | **<0.05** | **0.003** | 1.000 | **0.003** |
| **Temporal MT** (µm) | 360.7±7.0 | 327.8±6.9 | 328.5±7.0 | **<0.05** | **0.004** | 1.000 | **0.003** |
| ChT=choroidal thickness; CVI=choroidal vascularity index; MT=macular thickness. | | | | | | | |
